# YAP Promotes Chemoresistance to 5-FU in Colorectal Cancer Through mTOR/GLUT3 Axis

**DOI:** 10.7150/jca.100179

**Published:** 2024-11-04

**Authors:** Qixuan Xu, Zhesi Jin, Zhen Yuan, Zhiyuan Yu, Jingwang Gao, Ruiyang Zhao, Hanghang Li, Huiguang Ren, Bo Cao, Bo Wei, Linhua Jiang

**Affiliations:** 1 Department of General Surgery, The First Affiliated Hospital of Soochow University, Suzhou, Jiangsu, China.; 2 Department of General Surgery, First Medical Center, Chinese PLA General Hospital, Beijing, China.; 3 Medical School of Chinese PLA, Beijing, China.; 4 Department of Gastrointestinal Surgery, Affiliated Hospital of Jiangsu University, Zhenjiang, Jiangsu,China.; 5 School of Medicine, Nankai University, Tianjin, China.; 6 Department of General Surgery, Linfen Central Hospital, Linfen, Shanxi, China.

**Keywords:** YAP, chemoresistance, colorectal cancer, glycolysis, mTOR pathway, GLUT3.

## Abstract

**Background:** Although chemoresistance constitutes a significant barrier to the effectiveness of chemotherapy in colorectal cancer (CRC), its precise mechanisms remain unclear. YAP functions as an oncogene in various malignancies. However, the relationship between YAP and chemoresistance in CRC needs clarification.

**Methods:** The expression level of YAP in CRC tissues was assessed through immunohistochemistry (IHC), and the impact of YAP on CRC cell chemoresistance was evaluated using the Cell Counting Kit-8, EdU, and flow cytometry assays. Meanwhile, tumor proliferation was assessed *in vivo* by analyzing the expression of PCNA and Ki-67 in subcutaneous tumors via IHC. In addition, the TUNEL assay was employed to evaluate tumor apoptosis levels and western blot was utilized to detect the mTOR/GLUT3 pathway-related protein expression to provide insights into the underlying mechanism.

**Results:** YAP was highly expressed in CRC tissues and correlated with patient prognosis and clinicopathological features. Bioinformatic analysis based on the TCGA database revealed that YAP was associated with DNA replication, glycolysis, and the mTOR pathway. Meanwhile, YAP could enhance chemoresistance and glycolysis in CRC cells both *in vitro* and *in vivo*. Additional mechanistic experiments unveiled that YAP promoted CRC cell chemoresistance via the mTOR/GLUT3 axis.

**Conclusion:** This study validated the role of YAP as an oncogene in CRC, as it promoted chemoresistance through the mTOR/GLUT3 axis. These results suggested YAP as a potential target for promoting the efficacy of chemotherapy in patients with CRC.

## Introduction

Colorectal cancer (CRC) is one of the most prevalent malignant tumors of the digestive system. Recent global data underscore its prominence: it ranks third in incidence and second in mortality among all malignant tumors, next to only lung cancer 1. Although surgery remains the primary treatment modality for CRC, the lack of early diagnostic markers often leads to diagnoses at an advanced stage, depriving many patients of surgical intervention 2. The efficacy of 5-fluorouracil (5-FU), an important component of adjuvant therapy in patients with inoperable CRC, is significantly limited because of the development of chemoresistance 3. Thus, it is of utmost significance to delve into the molecular mechanisms of chemoresistance and devise strategies to enhance CRC cell sensitivity to 5-FU treatment and thereby improve overall survival.

The Hippo pathway, which is intricately involved in tumor initiation, progression, metastasis, and chemoresistance, has emerged as a critical player in these complex processes 4. YAP, a downstream effector in the Hippo pathway, is centrally involved in these processes, as the nuclear entry of unphosphorylated YAP regulates downstream gene expression, thereby serving as a critical node for the biological functions of the Hippo pathway 5, 6. YAP, in an oncogenic role, participates in various malignancies and affects cellular processes and tissue-level dynamics. For example, MEKK3 in human pancreatic cancer cells could stimulate epithelial-mesenchymal transition and extend survival in a pancreatic cancer mouse model via YAP regulation 7. At the tissue level, YAP expression was increased in the tumor tissues of patients with esophageal squamous cell carcinoma and led to poor prognosis, particularly concerning the N stage of these patients 8. Additionally, studies have highlighted the contribution of YAP to chemoresistance in small cell lung cancer, indicating that it is a potential target for treating chemoresistant small cell lung cancer 9. Moreover, YAP expression was closely related to doxorubicin efficacy in breast cancer 10. While previous research demonstrated that YAP plays a vital role in the promotion of CRC cell proliferation and metastasis *in vitro* 11, the relationship between YAP and chemoresistance in CRC remains unclear and needs further investigation.

Glucose transporters (GLUTs), specialized transport proteins that facilitate glucose entry into cells, maintain the energy required for tumor cell metabolism. Of note, the expression of GLUT3, closely linked to the EGFR signaling pathway in lung adenocarcinoma, is increased in various malignancies, including CRC 12-14. Although previous research suggested that the expression level of GLUT3 in CRC tissues is associated with the prognosis of patients 15, the specific mechanism of YAP and GLUT3 in regulating the biological behavior of CRC cells was not explored. Thus, this aspect needs to be explored.

The mammalian target of rapamycin (mTOR), which is known to form two distinct complexes (mTORC1 and mTORC2), is aberrantly activated in multiple cancers. The involvement of the PI3K/AKT/mTOR pathway in malignant tumor behaviors such as proliferation, migration, and stemness has been well documented 16-18. Indeed, targeting the mTOR pathway has shown promise in cancer treatment in both research and clinical settings 19-21. Recent research has established the close relationship among the mTOR pathway, glycolysis, and GLUT3 expression in tumor cells 22. However, the intricate interplay among YAP, the mTOR pathway, and glycolysis requires further investigation.

The present study findings indicated heightened YAP expression in CRC tissues, which correlated with patient prognosis and clinicopathological features. In addition, *in vitro* and *in vivo* experiments confirmed the inhibitory effect of YAP on the therapeutic efficacy of 5-FU in chemoresistant CRC cells. Mechanistically, YAP enhanced 5-FU-resistant CRC cells by activating the mTOR pathway to promote GLUT3 expression. Thus, inhibiting the YAP/mTOR/GLUT3 axis in conjunction with 5-FU may be a promising treatment strategy for patients with CRC.

## Materials and methods

### Clinical tissue specimens

One hundred and ten patients who underwent radical CRC resection at the Department of Gastrointestinal Surgery of the First Affiliated Hospital of Soochow University from 2014 to 2016 were randomly included retrospectively. Inclusion criteria are as follows: (1) The primary site of the tumor is the colon or rectum, with postoperative pathological diagnosis confirming colorectal adenocarcinoma; (2) Patients have undergone radical resection of colorectal cancer, encompassing complete excision of the tumor mass and a thorough dissection of surrounding lymph nodes; (3) Patients have not received neoadjuvant chemoradiotherapy or targeted therapy as adjuvant treatment prior to surgery. Exclusion criteria include: (1) Postoperative pathological diagnosis reveals signet-ring cell carcinoma, mucinous adenocarcinoma, or any other pathological subtypes; (2) Patients who have undergone neoadjuvant therapy before surgery. The primary tumor tissues and adjacent normal tissues, located >2 cm from the tumor edge, were resected. Colorectal adenocarcinoma was diagnosed by two independent pathologists. Written informed consent was obtained from all enrolled patients, and the study was approved by the Ethics Committee of the First Affiliated Hospital of Soochow University (2020076).

### Cell culture

The human CRC cell lines SW480, LOVO, HCT116, SW620, and HT29 were purchased from the Cell Bank of the Chinese Academy of Sciences. Complete medium was prepared with 10% fetal bovine serum and 1% penicillin-streptomycin. The RPMI-1640 complete medium was used for cell culture. Meanwhile, 5-FU-resistant HCT116 (HCT116R) and SW480 (SW480R) cells were obtained by increasing the 5-FU concentration from 0.01 to 80 μM for 6 months. All cells were cultured at 37℃ in a constant temperature incubator with 5% CO_2_.

### Cell transfection

Overexpression plasmids, shRNA, and negative control plasmids designed and manufactured by GenePharma (Suzhou, China) were obtained. First, 5-FU-resistant CRC cells were seeded into six-well plates and transfected using Lipofectamine^TM^ 3000 (Invitrogen, USA) on the second day at 80% confluence according to the manufacturer's instructions. Then, to establish cell lines exhibiting stable interference with YAP expression, a lentiviral packaging kit was utilized to cotransfect HEK-293T cells with both the packaging vectors and specialized interference plasmids. The shRNA and negative control sequences are presented in Supplementary Table 1.

### Quantitative real-time PCR (qRT-PCR)

Total RNA from cells was extracted using the TRIzol reagent (Thermo Fisher Scientific, USA) according to the manufacturer's instructions. After NanoDrop2000 quantification, samples with purity between 1.8 and 2.0 were used for subsequent experiments. RNA was reverse-transcribed into cDNA using the HiScriptIIQ Select RT Super Mix with random primers (Vazyme, China) for qPCR. The TB Green Premix Ex Taq II (Takara, Japan) kit was used for the subsequent PCR reaction. The 20-µL reaction system contained 10 µL TB Green Premix Ex Taq II, 0.8 µL forward primer (10 µM), 0.8 µL reverse primer (10 µM), 0.4 µL ROX reference dye, and template cDNA, which were mixed for amplification on ABI7500 (Applied Biosystems, USA). GAPDH served as the internal control, and the 2^-ΔΔct^ method was used to assess the expression of target genes. The relevant primer sequences are provided in Supplementary Table 2.

### Western blot

Cell and tissue proteins were extracted using RIPA containing 1% PMSF. After quantification using the BCA method (Beyotime, China), the proteins were separated using SDS-polyacrylamide gel electrophoresis and subsequently transferred to polyvinylidene fluoride membranes. The membranes were blocked with 5% skimmed milk for 2 h at room temperature to prevent nonspecific binding. Subsequently, the membranes were incubated with a primary antibody overnight at 4℃ and then with an HRP-conjugated antibody for 1 h at room temperature. Protein bands were visualized using an ECL western blotting substrate (Beyotime, China). The antibodies used for western blot are listed in Supplementary Table 3.

### Cell Counting Kit-8 (CCK-8) assay

The cell proliferation capacity was assessed using the CCK-8 assay. In brief, 3000 CRC cells were seeded in 96-well plates. At 24, 48, 72, and 96 h, 10 µL CCK-8 reagent (Vazyme, China) was added to each well and incubated for 1 h at 37℃ in the dark. Finally, absorbance was measured at 450 nm using a microplate reader.

### EdU assay

First, a 100 μL suspension containing 2 × 10^4^ CRC cells was seeded into a 96-well plate. Next, proliferating cells were stained using the EdU Image Kit (Abbkine, China) and 4',6-diamino-2-phenylindole according to the kit instructions. The stained cells were observed and enumerated under an inverted fluorescence microscope.

### Colony formation assay

Chemoresistant CRC cells were resuspended and seeded in six-well plates at 1000 cells per well. After 10 days of continuous culture, the cells were fixed with 4% paraformaldehyde and stained with crystal violet. Photographs were captured and the number of colonies were counted.

### Flow cytometry

Flow cytometry was employed to detect apoptosis in CRC cells. The Muse Annexin V & Dead Cell Kit (Luminex, USA) was utilized to stain the markers of the apoptotic process as per the manufacturer's instructions.

### Immunohistochemistry (IHC)

The human and mouse tissues were embedded in paraffin and fixed and sectioned. The antibodies used for IHC are listed in Supplementary Table 3. IHC staining was performed using the Metal Enhanced DAB Substrate Kit (Solarbio, China) according to the manufacturer's protocol. Staining intensity scores ranging from 0 to 3 represented negative, weak, moderate, and strong staining intensities, respectively, whereas staining extent scores ranging from 0 to 4 represented 0%-5%, 6%-25%, 26%-50%, 51%-75%, and >75% positive cells, respectively. The final score was calculated by multiplying the staining intensity score with the staining extent score. A final score of <4 was considered negative expression and that of >4 was deemed positive expression.

### TUNEL assay

The TUNEL assay was used to evaluate apoptosis in subcutaneous tumor tissue using the One Step TUNEL Apoptosis Assay Kit (Beyotime, China) according to the manufacturer's instructions. A fluorescence microscope was used to observe dUTP labeled with the green fluorescent probe fluorescein (FITC).

### Glycolysis

The lactate, pyruvate, and ATP production as well as the glucose uptake rate were used to reflect cellular glycolysis levels. The Glucose Uptake Colorimetric Assay Kit, ATP Colorimetric Assay Kit, Lactate Assay Kit II, and Pyruvate Colorimetric Assay Kit (Biovision, CA, USA) were used to detect the above indicators as per the respective kit instructions. A microplate reader was used to measure absorbance according to the respective optimal wavelength.

### Xenograft tumors in mice

Xenograft tumors in nude mice were developed as described previously 23. Four-week-old male BALB/C nude mice were purchased from Shanghai SLRC Laboratory Animal Co., Ltd. (Shanghai, China). The mice were subjected to experiments after 1 week of adaptive growth in a specific pathogen-free environment. During adaptive growth, the animals had unlimited access to sterile water and food. The environment was maintained at a stable temperature of 18°C-22°C, humidity of 40%-70%, and light/dark cycle of 12/12 h. The mice were randomly divided into three groups: HCT116 (wild type [WT]), HCT116R, and HCT116R-KD. Next, 5 × 10^6^ treated HCT116 cells were injected subcutaneously into the right flanks of the animals. Approximately 2 weeks after the injection, mice in both groups received 50 mg/kg 5-FU via an intraperitoneal injection every 3 days. The subcutaneous tumor volume was measured every 3 days, and the tumors were extracted for weight measurement and subsequent experiments after 28 days.

### Bioinformatic analysis

To investigate YAP-related genes in CRC based on the TCGA database, the online platform LinkedOmics (https://www.linkedomics.org/admin.php) was employed. The following parameters were set: cancer type, colorectal adenocarcinoma (COADREAD); sample cohort, TCGA_COADREAD; data type, RNAseq; platform, Hiseq RNA; attribute, YAP1; and statistical method: Spearman correlation test. Subsequently, Gene Ontology (GO), Kyoto Encyclopedia of Genes and Genomes (KEGG) and Gene Set Enrichment Analysis (GSEA) were performed.

### Statistical analysis

All experiments were repeated three times. The SPSS 23.0, GraphPad Prism 8, and R software were employed for data analysis. Data are presented as means ± standard deviation, and Student's t-test (unpaired, two-tailed) was used to compare means between two groups. The IHC scores of YAP in CRC tumors and the adjacent normal tissues were analyzed using the Chi-squared test or Fisher's exact test. Finally, survival curves were generated using the Hiplot online website (https://hiplot-academic.com/). *P* < 0.05 indicated statistical significance.

## Results

### YAP is highly expressed in CRC tissues

**Figure 1:**
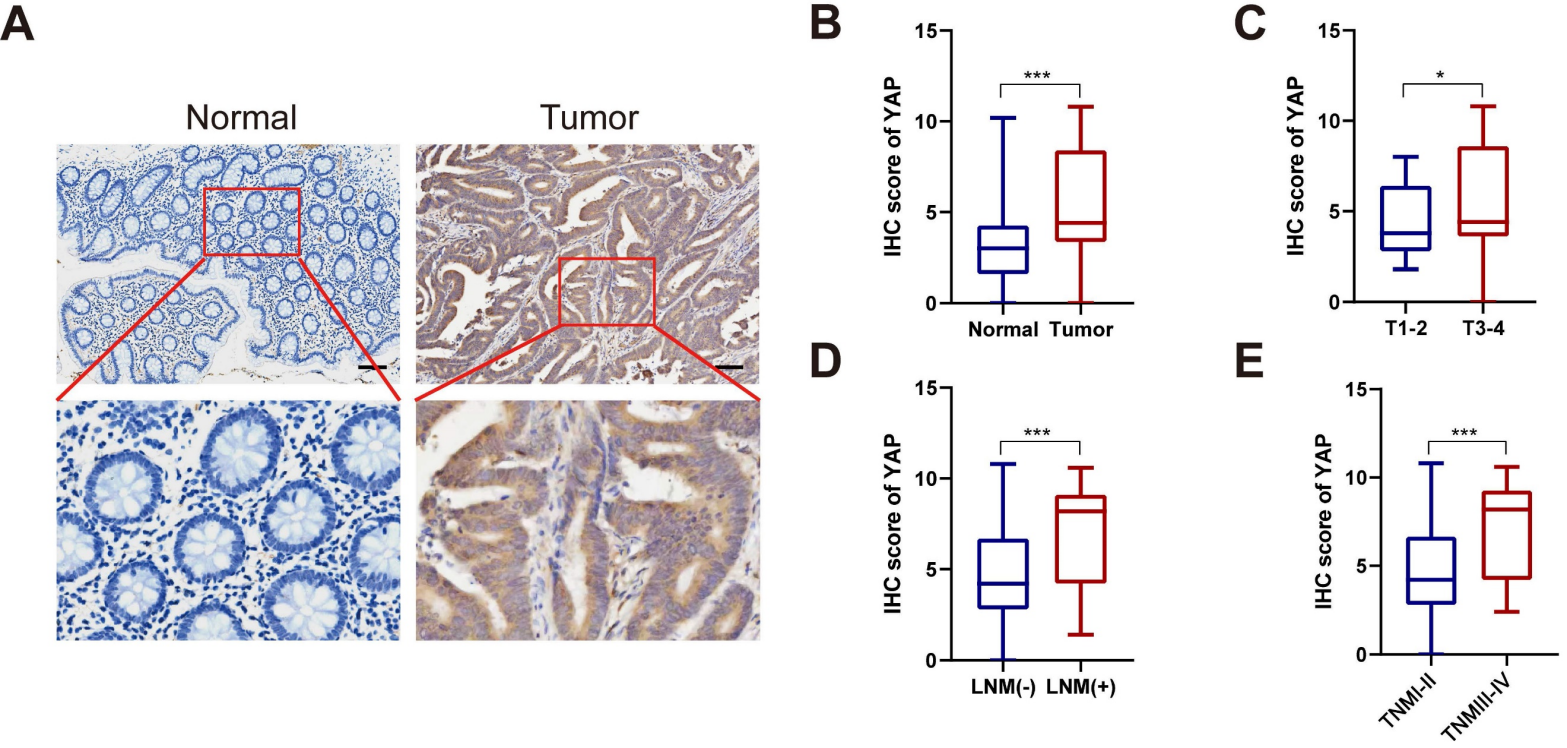
YAP expression in CRC tissues. **(A)** Representative IHC staining of YAP in CRC tissues and adjacent normal tissues. **(B)** IHC scores of YAP in CRC tissues and adjacent normal tissues. **(C)** IHC scores of YAP in T1-2 and T3-4 CRC tissues. **(D)** IHC scores of YAP in LNM (-) and LNM (+) CRC tissues. **(E)** IHC scores of YAP in TNM I-II and TNM III-IV CRC tissues.

To investigate YAP expression in CRC, tumor tissues and the adjacent normal tissues from 110 patients with CRC were retrospectively analyzed. YAP protein expression in CRC tissues was assessed via IHC. As illustrated in Figure 1A-B, YAP was abnormally upregulated in tumor tissues compared with the adjacent normal tissues. In addition, subgroup analysis revealed elevated YAP levels in the tumor tissues of patients with T stage 3-4, lymph node metastasis, and TNM stage III-IV compared with patients with T stage 1-2, no lymph node metastasis, and TNM stage I-II (Figure 1C-E). This finding suggested a potential correlation between YAP expression and CRC stage. Meanwhile, a chi-squared test used to analyze the relationship between the YAP expression and clinicopathological characteristics of patients with CRC indicated that YAP expression correlated with lymph node metastasis, venous invasion, and TNM staging but not with age, gender, tumor size, depth of tumor invasion, degree of differentiation, and neural invasion (Table 1).

### YAP expression is associated with prognosis

**Figure 2:**
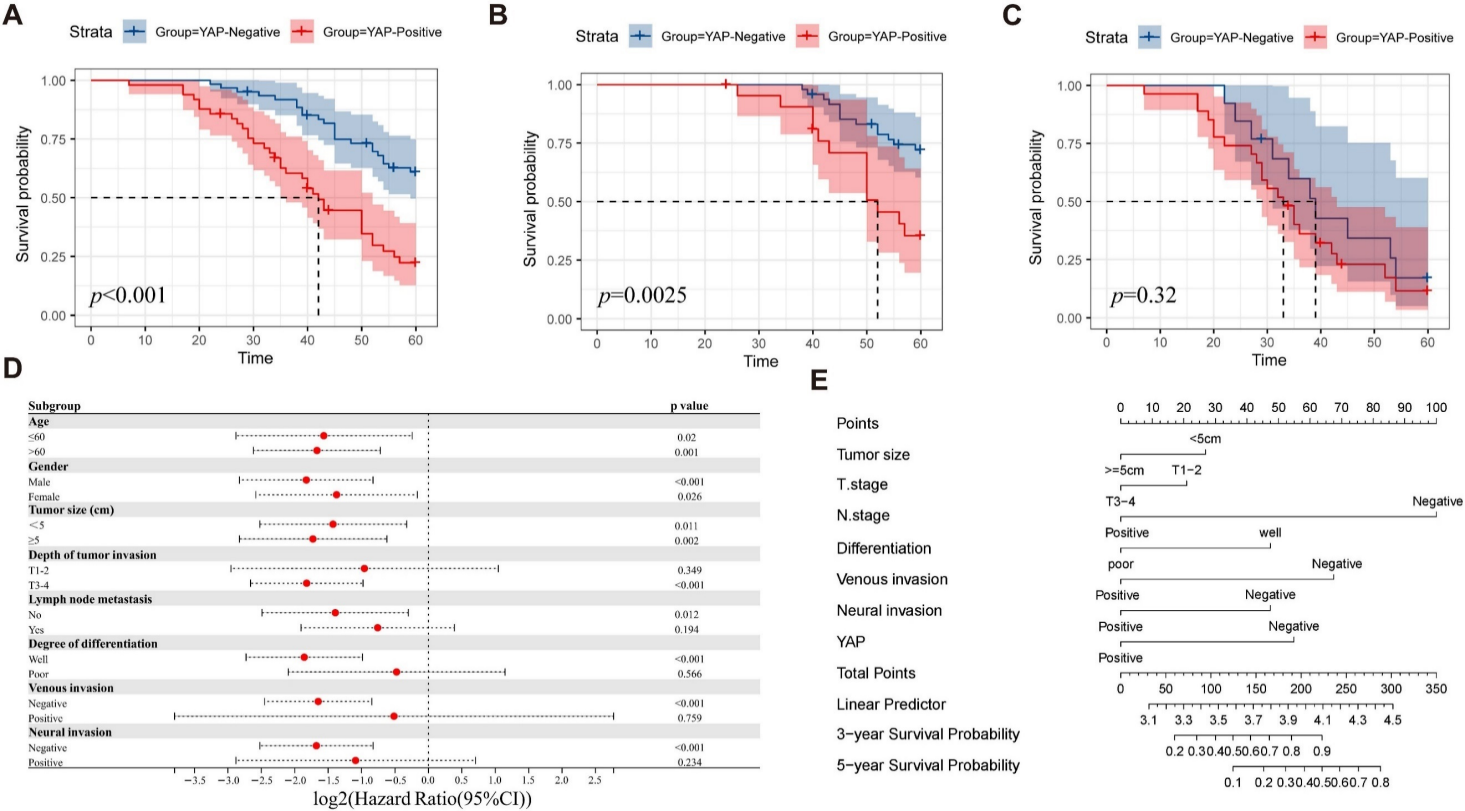
Correlation between YAP and prognosis of patients with CRC. Overall survival analysis **(A)** shows significantly lower survival in patients with CRC with positive YAP expression, particularly in TNM stage I-II **(B)** compared with TNM stage III-IV **(C)**. Subgroup analysis **(D)** identifies factors that influence survival. Nomogram (E) predicts the survival of patients with CRC.

To evaluate the impact of YAP expression on patient prognosis, the patients were divided into YAP positive and negative groups based on their IHC scores. The Kaplan-Meier survival analysis demonstrated significantly reduced survival time in the positive group compared with the negative group (Figure 2A). In addition, subgroup analysis indicated that YAP expression was associated with the prognosis of patients with TNM stage I-II but not with TNM stage III-IV (Figure 2B-C). This result suggested that YAP can be used as a prognostic marker for patients with CRC, particularly for those with early stage disease. Meanwhile, univariate analysis revealed that the tumor size, depth of tumor invasion, lymph node metastasis, degree of differentiation, venous invasion, neural invasion, and YAP expression were the independent risk factors of prognosis. Multivariate analysis confirmed that lymph node metastasis, venous invasion, and YAP expression were strongly associated with prognosis (Table 2). To better identify the effect of YAP expression level on the prognosis of patients with CRC, the patients were categorized according to the clinicopathological characteristics and a Cox regression model was used to predict the relationship between YAP and prognosis. The results showed that YAP positivity could predict the survival of patients with CRC, regardless of age, gender or tumor size (Figure 2D). In addition, YAP was associated with poor survival in patients with CRC with T3-4, negative lymph node metastasis, well differentiation, negative venous invasion, and negative neural invasion (Figure 2D). For a comprehensive evaluation of the effect of YAP expression on prognosis, a nomogram model was constructed, which considered various clinicopathological characteristics. The model revealed that YAP plays a crucial role in predicting three-year and five-year survival rates in CRC patients (Figure 2E).

### YAP is related to DNA replication and mTOR pathways

**Figure 3:**
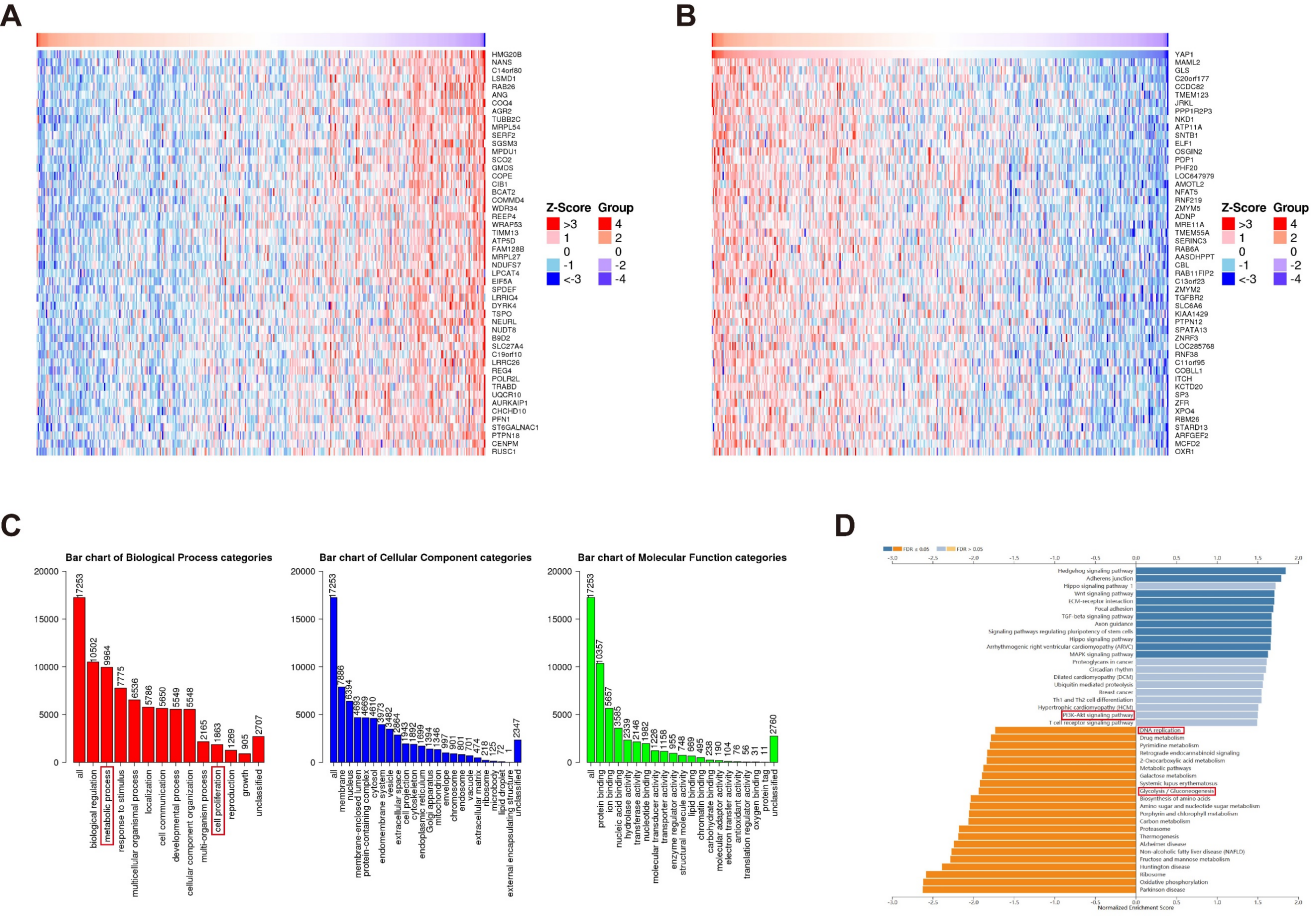
Enrichment analysis of YAP functions in CRC. The top 50 genes positively (A) and negatively (B) associated with YAP in CRC are shown. GO enrichment analysis (C) illustrates the correlation of identified genes with biological processes (BP), cellular components (CC), and molecular functions (MF). KEGG analysis (D) indicates YAP involvement in various regulatory pathways.

Although YAP has emerged as a potential diagnostic and prognostic marker of CRC, the underlying mechanisms remain unclear. Utilizing the TCGA database, genes positively and negatively correlated with YAP in CRC were screened using LinkedOmics (Figure 3A-B). The subsequent GO enrichment analysis of these genes revealed associations with cell proliferation and metabolic processes, suggesting that YAP may be considered an oncogene that regulates cell metabolism (Figure 3C). The KEGG analysis demonstrated that the YAP-related genes were enriched in the DNA replication and PI3K/AKT/mTOR pathways (Figure 3D). mTOR pathway activation has been shown to induce improper DNA replication in various cancer cells, which contributes to drug resistance 23. Thus, it was hypothesized that YAP, acting as an oncogene, is involved in CRC cell drug resistance.

### YAP promotes 5-FU resistance in CRC cells

**Figure 4:**
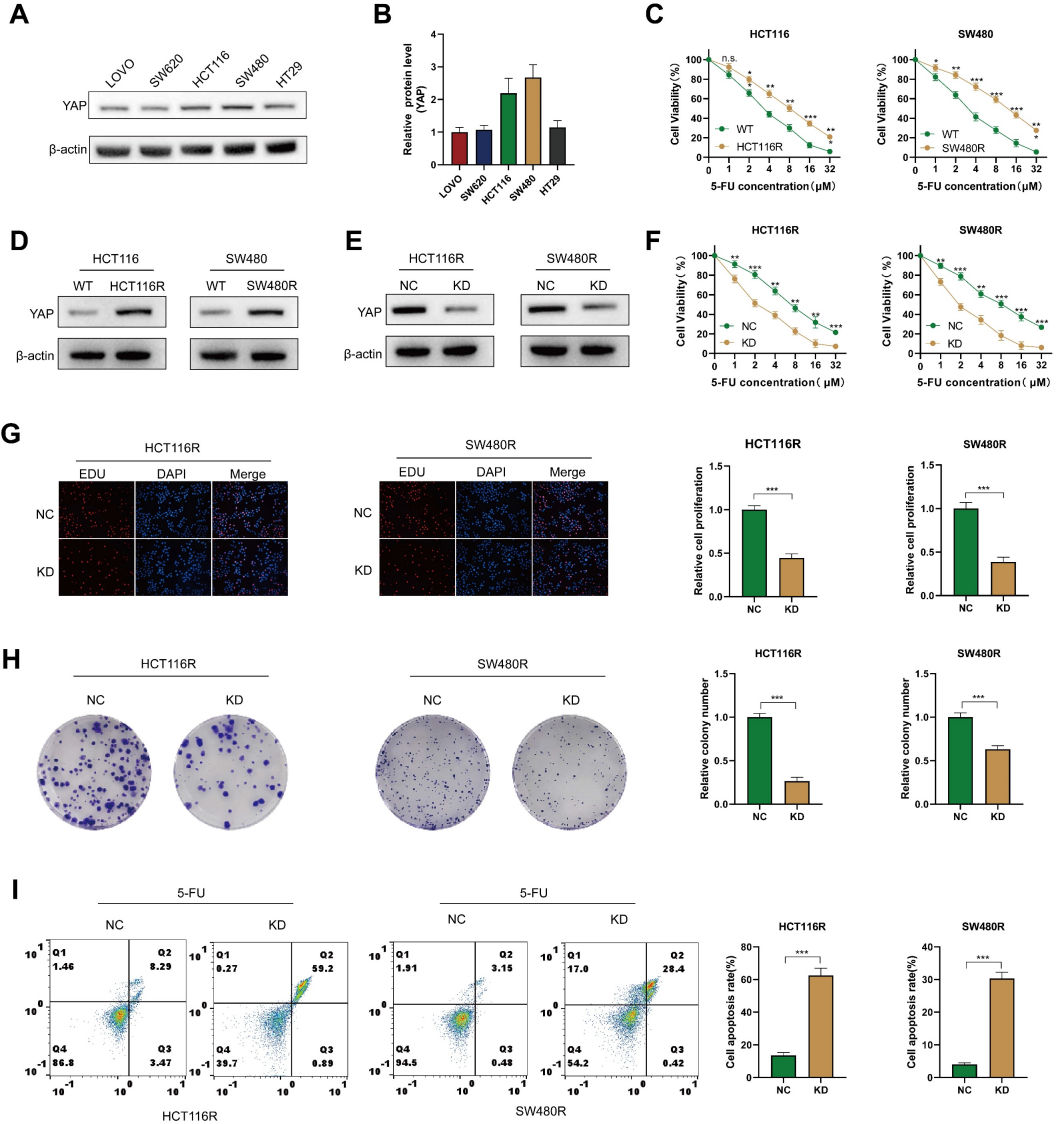
Role of YAP in the chemoresistance of CRC cells. **(A-B)** Western blot analysis of YAP expression in LOVO, SW620, HCT116, SW480, and HT29 cell lines. **(C)** Viability of HCT116 and SW480 cells in WT and chemoresistant cells. **(D)** Western blot analysis of YAP expression in HCT116R and SW480R cells. **(E)** Western blot analysis of YAP expression in the NC and KD groups. **(F)** Viability of HCT116R and SW480R cells in the NC and KD groups. **(G)** EdU assay was used to measure the proliferation of chemoresistant cells in the NC and KD groups. **(H)** Colony formation assay was utilized to measure the proliferation of chemoresistant cells in the NC and KD groups. **(I)** Flow cytometry analysis was performed to analyze the apoptosis of chemoresistant cells in the NC and KD groups.

To explore the role of YAP in CRC chemoresistance *in vitro*, YAP expression was assessed in different CRC cell lines via western blot. The results revealed that HCT116 and SW480 exhibited the highest YAP expression. Therefore, these cell lines were selected for subsequent experiments (Figure 4A-B). First, 5-FU-resistant CRC cells (including HCT116R and SW480R) were produced, and increased resistance to 5-FU was observed in these cells groups compared with cells in the WT groups (Figure 4C). In addition, western blot analysis revealed elevated YAP expression in chemoresistant HCT116R and SW480R cells (Figure 4D). To investigate the impact of YAP on chemoresistance, YAP expression was stably knocked down in chemoresistant cells using siRNA, which was verified via western blot (Figure 4E). The CCK-8 assay demonstrated that YAP knockdown significantly enhanced the effect of 5-FU on chemoresistant cells (Figure 4F). Moreover, the EdU and colony formation assays revealed an increased proliferation of YAP-knockdown chemoresistant cells (Figure 4G-H) and flow cytometry revealed that YAP inhibited apoptosis in chemoresistant cells (Figure 4I). These results affirmed that YAP promoted chemoresistance in CRC cells *in vitro*.

### Downregulation of YAP reprograms glycolysis in chemoresistant CRC cells

**Figure 5:**
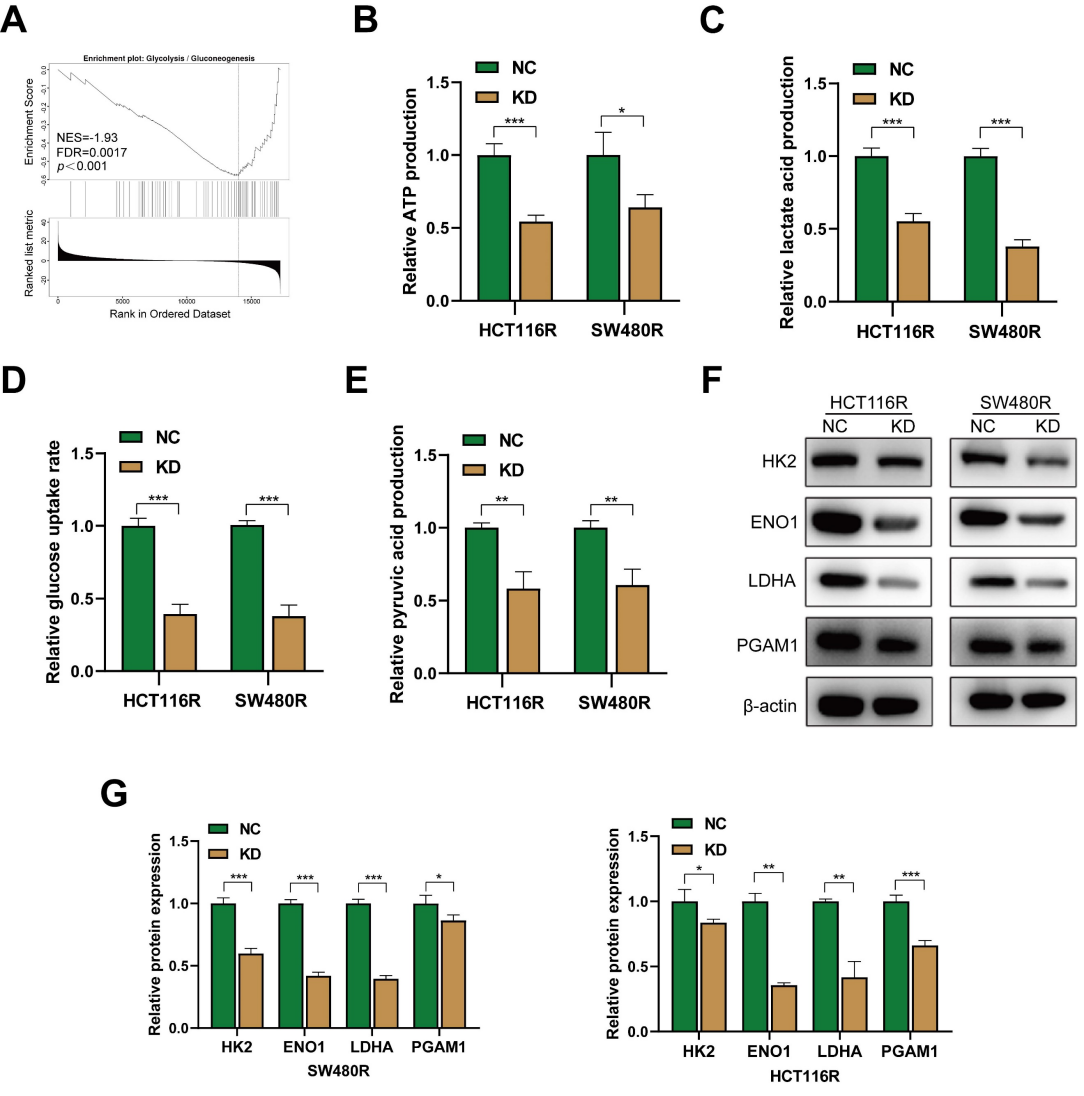
The effect of YAP in CRC cells glycolysis. **(A)** GSEA of gene sets for glycolysis signaling pathway. **(B-E)** ATP production **(B)**, lactate production **(C)**, relative glucose uptake rate **(D)**, and pyruvate production **(E)** of chemoresistant cells in the NC and KD group. **(F)** Western blot analysis of HK2, ENO1, LDHA, and PGAM1 expression in chemoresistant cells. **(G)** Quantification of the expression of the abovementioned proteins.

The KEGG enrichment results indicated that YAP-related genes in CRC were primarily enriched in the glycolysis pathway. GSEA results revealed that YAP was associated significantly with the glycolysis pathway (Figure 5A). Thus, it was hypothesized that YAP influences chemoresistance in CRC cells by modulating glucose metabolism. To evaluate this, a series of glycolytic assays were performed to examine the effect of YAP on the glycolysis levels of chemoresistant CRC cells. YAP inhibition significantly reduced the lactate, pyruvate, and ATP production and decreased glucose uptake rate in chemoresistant CRC cells (Figure 5B-E). In addition, western blot showed that YAP knockdown significantly inhibited the expression of glycolysis-related genes in chemoresistant CRC cells (Figure 5F-G). Taken together, YAP participated in the glycolytic reprogramming of chemoresistant CRC cells.

### YAP promotes 5-FU resistance *in vivo*

**Figure 6:**
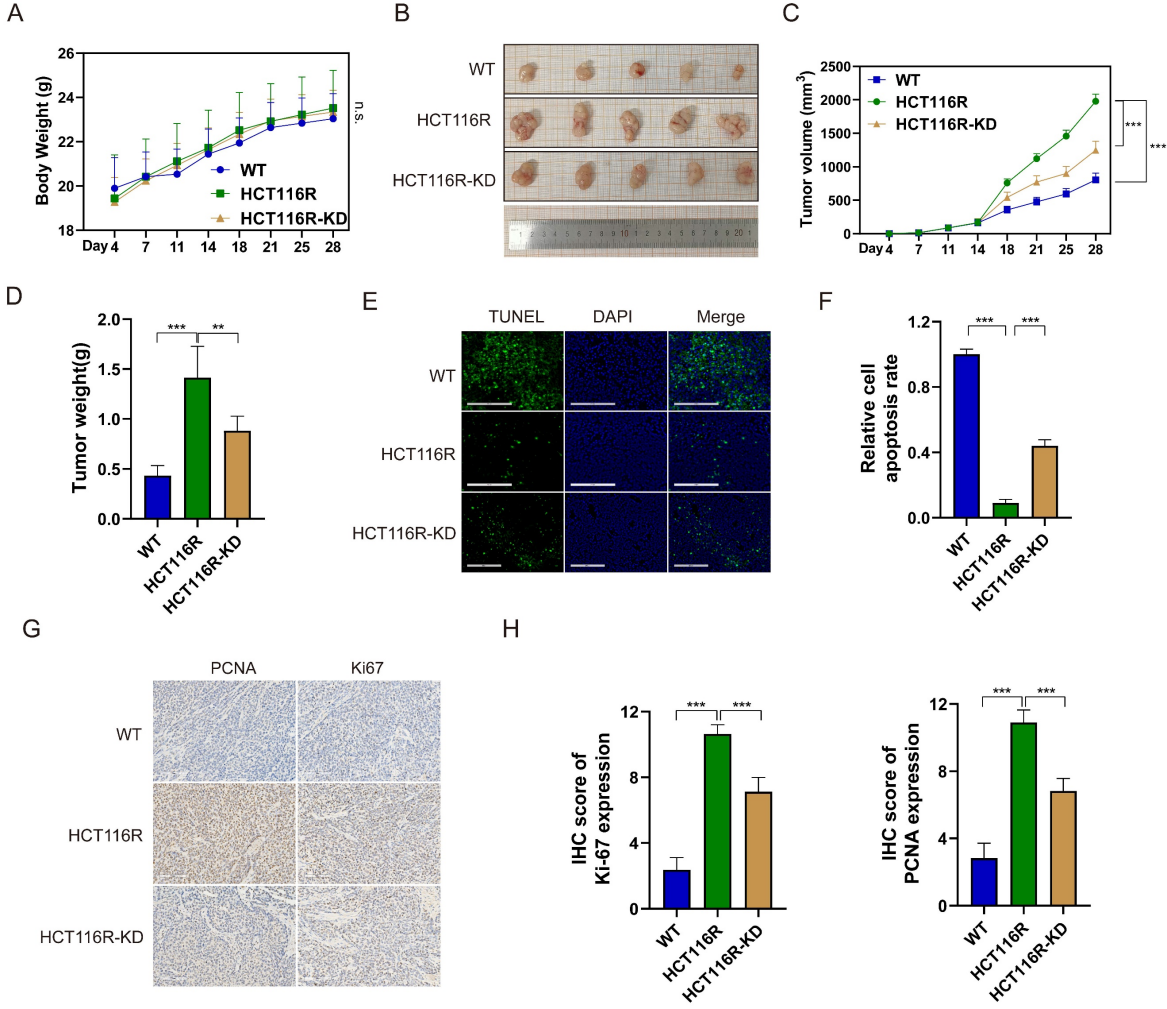
Effect of YAP knockdown on the chemoresistance of CRC cells *in vivo*. **(A)** Mouse body weight in the WT, HCT116R, and HCT116R-KD groups. **(B)** Representative images of subcutaneous tumors. **(C)** Tumor volume in the WT, HCT116R, and HCT116R-KD groups. **(D)** Analysis of tumor weight. **(E-F)** TUNEL assay was performed to measure the apoptosis level in tumors. **(G-H)** IHC staining of PCNA and Ki67 in subcutaneous tumors.

To assess the impact of YAP on 5-FU efficacy in CRC cells *in vivo*, subcutaneous tumor xenograft models were established. The mice were randomly divided into the three groups of HCT116 (WT), HCT116R, and HCT116R-KD and injected with HCT116, HCT116R, and HCT116R cells transfected with sh-YAP, respectively. All mouse groups received intraperitoneal 5-FU from day 7 after injection. No significant difference in the body weight was observed among the three groups (Figure 6A). However, tumor volume and weight were significantly increased in the HCT116R group compared with the WT group, with YAP knockdown reversing this trend (Figure 6B-D). The TUNEL assay confirmed that YAP knockdown significantly increased 5-FU-induced apoptosis in subcutaneous tumors (Figure 6E-F). Likewise, the IHC staining of subcutaneous tumors revealed increased PCNA and Ki67 positive cells in the HCT116R-KD group compared with the HCT116R group (Figure 6G-H). These findings indicated that YAP downregulation could reverse CRC cell chemoresistance* in vivo*.

### YAP regulates 5-FU resistance through GLUT3 in CRC cells

**Figure 7:**
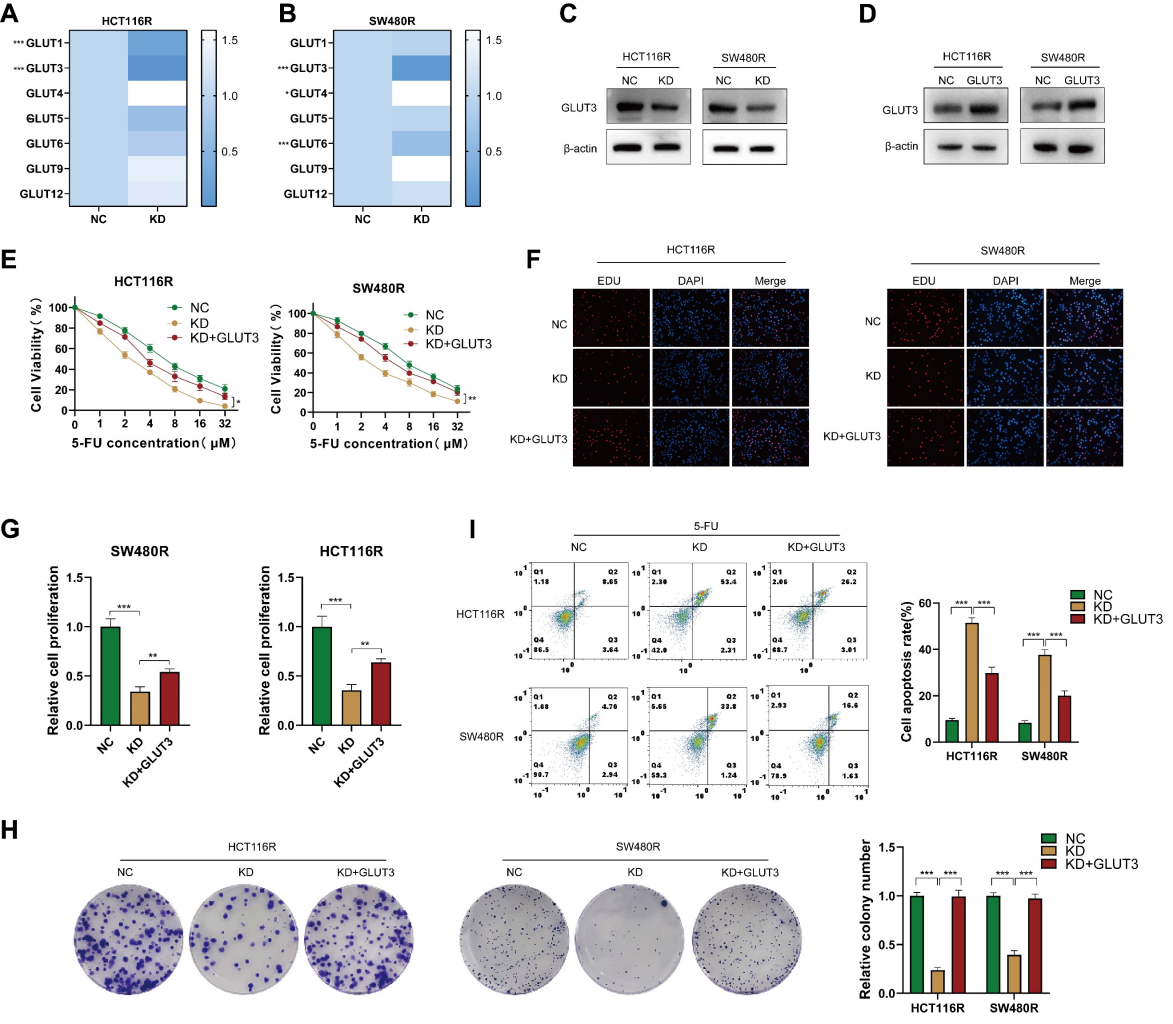
Role of the YAP/GLUT3 axis in CRC chemoresistance. **(A-B)** qPCR assay was used to measure the expression of GLUTs in chemoresistant cells in the NC and KD groups. **(C)** Western blot was performed to assess GLUT3 expression in the NC and KD groups. **(D)** Western blot was used to measure GLUT3 expression in the NC and GLUT3 groups. **(E)** Viability of HCT116R and SW480R cells in the NC, KD, and KD + GLUT3 groups. **(F-G)** EdU assay was employed to measure the proliferation of chemoresistant cells in the abovementioned groups. **(H)** Colony formation assay was performed to measure the proliferation of chemoresistant cells in the abovementioned groups. **(I)** Flow cytometry was used to analyze apoptosis in chemoresistant cells in the abovementioned groups.

The abnormal expression of GLUT has been shown to affect chemotherapy by regulating glycolysis 24, 25. The examination of GLUT family mRNA levels in chemoresistant CRC cells after YAP downregulation revealed that GLUT3 was the most significantly decreased member in both HCT116R-KD and SW480R-KD cells (Figure 7A-B). Meanwhile, GLUT2, GLUT7, GLUT8, GLUT10, and GLUT11 were undetected. Western blot confirmed the role of YAP in promoting GLUT3 expression at the protein level (Figure 7C). Thus, it was hypothesized that the effect of YAP on CRC cell chemoresistance is GLUT3-dependent. To confirm this, a GLUT3 stably overexpressing cell line was generated in chemoresistant CRC cells; the overexpression was verified using western blot (Figure 7D). The CCK-8, EdU, colony formation, and flow cytometry assays demonstrated that YAP knockdown increased the sensitivity of chemoresistant CRC cells to 5-FU, which was reversed by the overexpression of GLUT3 (Figure 7E-I). These findings suggested that YAP promotes CRC cell chemoresistance through GLUT3-mediated glycolytic reprogramming.

### YAP promotes CRC cell chemoresistance through mTOR/GLUT3 pathway

**Figure 8:**
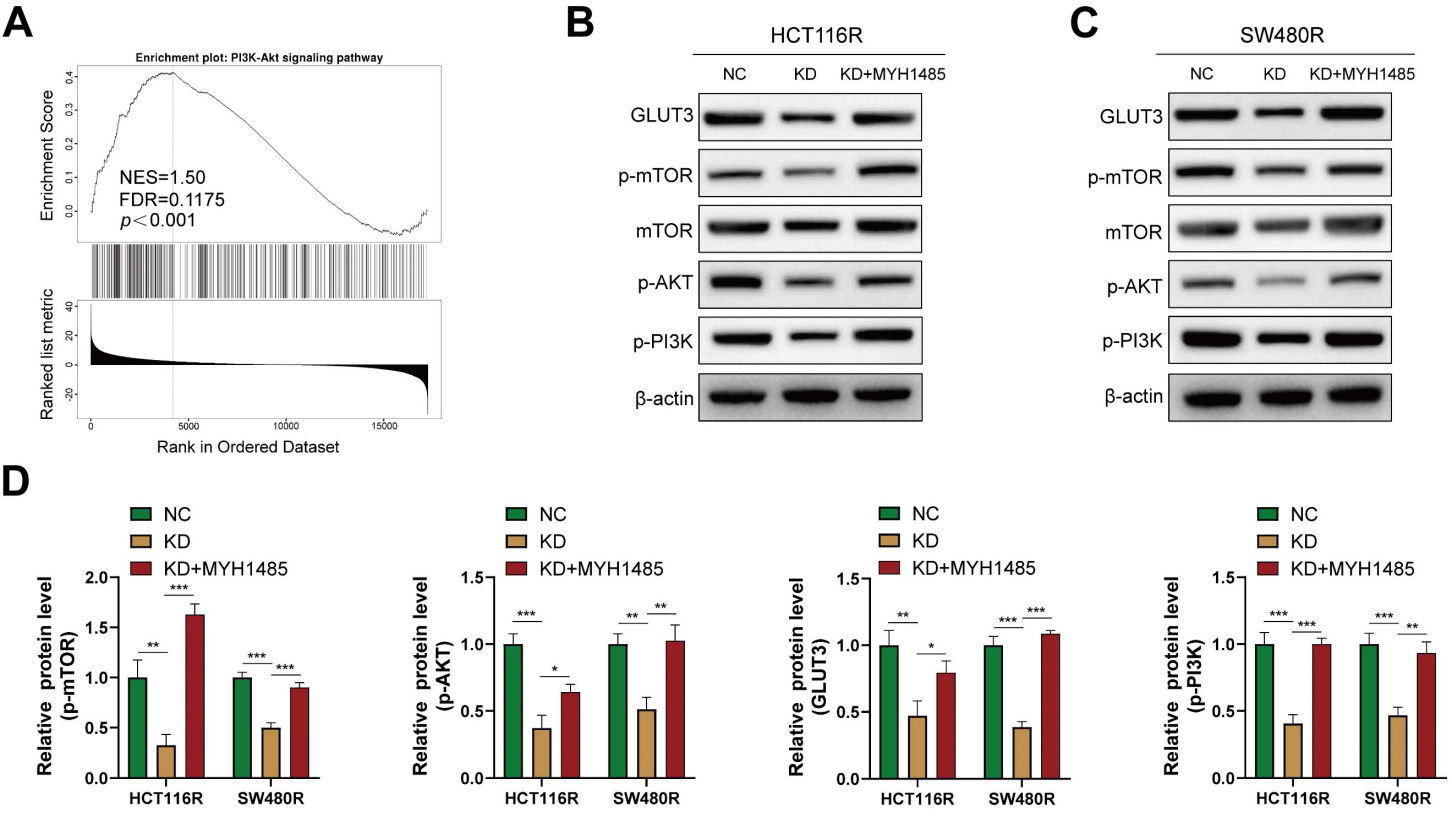
Effect of YAP on the mTOR pathway **(A)** GSEA of gene sets for the PI3K/AKT signaling pathway. **(B-C)** Western blot analysis of GLUT3, p-mTOR, mTOR, p-AKT, and p-PI3K expression in chemoresistant cells. **(D)** Quantification of the expression of the abovementioned proteins.

The activation of the mTOR signaling pathway is associated with glycolysis and drug resistance in various cancers 26, 27. In this context, previous studies have reported that YAP can activate the PI3K/AKT/mTOR pathway 28. However, the relationship between mTOR and GLUT3 in CRC remains unclear. GSEA performed on the aforementioned YAP-related genes revealed a significant correlation between YAP and the mTOR pathway (Figure 8A). Thus, it was hypothesized that YAP promotes GLUT3 expression by activating the mTOR pathway, thereby enhancing CRC cell chemoresistance. Western blot results indicated that the inhibition of YAP in chemoresistant CRC cells decreased the expression of p-mTOR, p-PI3K, and p-AKT, which are key proteins in the mTOR pathway, without significantly altering the total mTOR expression (Figure 8B-D). However, treatment with MHY1485, an mTOR pathway activator, increased GLUT3 expression, a response suppressed by YAP downregulation (Figure 8B-D). These findings suggested that YAP promotes GLUT3 expression by activating the mTOR pathway in CRC cells, thereby augmenting CRC chemoresistance.

## Discussion

For most patients with advanced CRC, surgery combined with adjuvant chemoradiotherapy has become the standard treatment 29-31, as this combination can significantly improve the recurrence rate and prognosis in these patients 32-35. However, the development of chemoresistance has become a major obstacle in the progress of CRC treatment 36. In the present study, YAP was identified as an oncogene that promoted chemoresistance in CRC cells both *in vitro* and *in vivo*. Therefore, it may be used as a diagnostic and prognostic marker of CRC.

YAP, a key downstream effector of the Hippo pathway, has been implicated in promoting proliferation and metastasis in various cancers 37-40. Indeed, some recent studies have shown that YAP is highly expressed in various cancer tissues. For example, Pei *et al.* showed that YAP expression was higher in cholangiocarcinoma tissues than in normal bile duct tissues and that patients with high YAP expression exhibited poor prognosis 41. In the context of CRC, the present study reaffirmed YAP overexpression in CRC tissues, which correlated with poor patient prognosis. Of note, YAP expression was as an independent risk factor of CRC prognosis and significantly predicted 3- and 5-year survival rates in patients with CRC. These findings are in line with existing research suggesting elevated YAP expression in CRC tissues and its association with unfavorable patient outcomes. For instance, Wang *et al.* detected higher YAP expression in CRC tumor tissues than in normal intestinal tissues, and the higher expression correlated with poor patient prognosis 42. These observations are consistent with the present study findings.

Although the mechanisms of chemoresistance in cancer cells remain unclear, YAP has been shown to promote chemoresistance in certain cancers. In breast cancer cells, YAP interacts with SRGN to regulate HDAC2 expression, thereby promoting breast cancer cell resistance to multiple drugs 40. Likewise, Zhou *et al.* reported abnormal YAP expression in chemoresistant hepatocellular carcinoma (HCC) cells, displaying its ability to induce drug resistance through the mTOR pathway 43. The functional enrichment analysis unveiled the association of YAP-related genes with the DNA replication and mTOR pathways, indicative of the potential involvement of YAP in CRC cell chemoresistance. Of note, *in vitro* and *in vivo* experiments substantiated the prospect of YAP inhibition in enhancing the efficacy of 5-FU on CRC cells, thereby positioning YAP as a promising therapeutic target for CRC.

KEGG enrichment analysis in the former part revealed that YAP was highly associated with the glycolysis pathway. The reprogramming of glucose metabolism is an important hallmark of malignancy, as it can provide energy for tumor cell proliferation 44. This feature may be exploited to improve cancer therapy via the inhibition of glucose metabolism 45. GLUT3, a member of the GLUT family, is highly expressed on various malignant tumor tissues, including CRC 14, 46. In the current study, YAP downregulation in chemoresistant CRC cells suppressed the glycolysis level as well as the expression of key proteins in the glycolysis pathway. Mechanistically, GLUT3 expression was significantly reduced in YAP-knockdown chemoresistant CRC cells. In addition, rescue experiments confirmed that YAP could promote CRC cell chemoresistance through GLUT3. Consistent with these findings, YAP has been shown to regulate FOXC2-induced glycolysis by upregulating HK2 expression in nasopharyngeal carcinoma 47. Likewise, in CRC, the YAP/GLUT3 axis could activate the metabolic reprogramming of CRC cells to promote tumor metastasis, indicating that YAP can regulate tumor malignancy and glucose metabolism in CRC 48.

The activation of the mTOR signaling pathway is the key to the promotion of cell growth, and its abnormal expression is often associated with several cancers 49-51. mTOR, a mediator of PI3K signaling pathway, is activated by the upstream PI3K/AKT 52, and the mTOR pathway is closely related to the development of chemoresistance. For example, studies have shown that the mTOR pathway is abnormally activated in oxaliplatin-treated CRC cells, and *in vitro* experiments have confirmed that the combination of oxaliplatin and mTOR inhibitors exhibits a positive synergistic effect 53. Based on GSEA, it was speculated that YAP is closely related to the mTOR pathway in CRC. To test this hypothesis, the expression of YAP was knocked down in CRC cells, which revealed that the expression of p-mTOR, p-PI3K, and p-AKT, which are key proteins in the mTOR pathway, was decreased while the total mTOR expression level did not change significantly. The mTOR agonist MYH1485 used to activate the mTOR pathway in CRC cells showed that the expression level of GLUT3 was upregulated, thereby validating the positive promotion effect of the mTOR pathway on GLUT3. In HCC cells, the inhibition of the mTOR pathway impeded the expression of GLUT1/3 and promoted the radiosensitivity of HCC cells 54. This is line with the present study finding that mTOR regulates GLUT3 expression.

Taken together, the current study results suggested YAP as a potential diagnostic and prognostic marker of CRC. In addition, the study uncovered the role of YAP in promoting chemoresistance in CRC cells. The involvement of YAP in glycolytic reprogramming, GLUT3 modulation, and mTOR pathway activation expands the understanding of the molecular mechanisms underlying CRC progression and resistance. Thus, targeting YAP may be a promising avenue for enhancing the efficacy of chemotherapy for CRC. The study findings offered valuable insights for future therapeutic strategies in CRC.

## Supplementary Material

Supplementary figures and tables.

## Figures and Tables

**Table 1:** Relationship between YAP and clinic-pathological factors in CRC patients.

	YAP		
	Negative	Positive	*P*
Age			0.235
≤60	28	17	
>60	33	32	
Gender			0.079
Male	40	24	
Female	21	25	
Tumor size (cm)			0.162
<5	38	24	
≥5	23	25	
Depth of tumor invasion			0.366
T1-2	17	10	
T3-4	44	39	
Lymph node metastasis			<0.001^*^
No	50	23	
Yes	11	26	
Degree of differentiation			0.122
Well	54	38	
Poor	7	11	
Venous invasion			0.024^*^
Negative	60	43	
Positive	1	6	
Neural invasion			0.309
Negative	56	42	
Positive	5	7	
TNM staging			<0.001^*^
I-II	48	22	
III-IV	13	27	

^*^p<0.05

**Table 2:** Results of univariate and multivariate analyses of postoperative patients' survival by Cox's proportional hazard model.

Characteristics	n	Univariate analysis			Multivariate analysis		
		HR	95% CI	*P*	HR	95% CI	*P*
Age (≤60/>60)	45/65	0.614	0.354-1.063	0.081			
Gender (Male/Female)	64/46	0.930	0.551-1.568	0.784			
Tumor size (<5/≥5)	62/48	0.556	0.331-0.932	0.026^*^	0.658	0.369-1.173	0.156
Depth of tumor invasion (T1-2/T3-4)	27/83	0.344	0.163-0.727	0.005^*^	0.668	0.282-1.585	0.361
Lymph node metastasis (Negative/Positive)	73/37	0.179	0.105-0.305	<0.001^*^	0.271	0.145-0.508	<0.001^*^
Degree of differentiation (Well/Poor)	92/18	0.436	0.235-0.810	0.009^*^	0.737	0.367-1.481	0.392
Venous invasion (Negative/Positive)	103/7	0.382	0.163-0.899	0.028^*^	3.341	1.028-10.853	0.045^*^
Neural invasion (Negative/Positive)	98/12	0.324	0.167-0.628	0.001^*^	0.413	0.168-1.018	0.055
YAP expression	70/40	0.317	0.186-0.540	<0.001^*^	0.383	0.215-0.682	0.001^*^

^*^p<0.05
