# Supplementary material for: YAP Promotes Chemoresistance to 5-FU in Colorectal Cancer Through mTOR/GLUT3 Axis
Source: J Cancer. 2024 Nov 4;15(20):6784–97. doi: 10.7150/jca.100179 (PMC11632981; doi:10.7150/jca.100179)
Supplement: Supplementary file 1 — Supplementary figures and tables. [file jcav15p6784s1.pdf]

Supplementary Table 1 Oligonucleotides used in this study

Definition	Sequence(5'-3')
sh-YAP	GGUCAGAGAUACUUCUUAAAU
sh-NC	UUCUCCGAACGUGUCACGUTT

Supplementary Table 2 Primers used in this study

Name	Primer	Sequence
GLUT1	Forward primer	ACTCCTCGATCACCTTCTGG
	Reverse primer	ATGGAGCCCAGCAGCAA
GLUT2	Forward primer	ATCCAAACTGGAAGGAACCC
	Reverse primer	CATGTGCCACACTCACACAA
GLUT3	Forward primer	GATGGGCTCTTGAACACCTG
	Reverse primer	GACAGCCCATCATCATTTCC
GLUT4	Forward primer	CCCCAATGTTGTACCCAAAC
	Reverse primer	CTTCCAACAGATAGGCTCCG
GLUT5	Forward primer	TGACAGCAGCCACGTTGTA
	Reverse primer	GCAACAGGATCAGAGCATGA
GLUT6	Forward primer	AACATGATGCTCAGCTTCCG
	Reverse primer	CTGACCTGCATCTGACCAAA
GLUT7	Forward primer	TGTTGTTGATCAGCAGGGTC
	Reverse primer	TGCTGCTTCTATGGTCTTGC
GLUT8	Forward primer	GAAGCACATGAGAAGCAGCA
	Reverse primer	CTGTGTGCAGCTAATGGTCG
GLUT9	Forward primer	GGTGCCTGCAATGATGAAG
	Reverse primer	GAGTATCGTGGGCATTCTGG
GLUT10	Forward primer	CAGCAAAGACACAGAGGCAC
	Reverse primer	GGAAAGTTTGTCCGGCG
GLUT11	Forward primer	AAACAGGATTGCTGCTGACA
	Reverse primer	CGTGTCTCTGTATCCCCTGG
GLUT12	Forward primer	ACGAGCCATGGCTTTAACTT
	Reverse primer	CATGGCAGGCCAATAAGAT
GAPDH	Forward primer	CAGGAGGCATTGCTGATGAT
	Reverse primer	GAAGGCTGGGGCTCATTT

Supplementary Table 3 Antibodies used in this study

Antibodies	Manufacturers	Applications
YAP	CST, 14074S	1:1000 for WB, 1:500 for IHC
PCNA	CST,13110S	1:1000 for WB, 1:10000 for IHC
Ki-67	Abcam, ab16667	1:1000 for WB, 1:200 for IHC
GLUT3	Abcam, ab314193<br>Proteintech, 20403-1-AP	1:1000 for WB

mTOR	Abcam, ab134903	1:1000 for WB
p-mTOR	Abcam, ab109268	1:1000 for WB
p-PI3K	CST, 17366S	1:1000 for WB
p-AKT	Abcam, ab38449	1:1000 for WB
HK2	CST, 2867S	1:1000 for WB
ENO1	CST, 3810S	1:1000 for WB
LDHA	CST, 3582S	1:1000 for WB
PGAM1	Abcam, ab288376	1:1000 for WB
β-actin	Servicebio, GB15003-100	1:1000 for WB
